# Training for the Delivery of a Comprehensive High‐Dose Aphasia Therapy Program via Telerehabilitation: Effectiveness and Satisfaction With the TeleCHAT Training Package

**DOI:** 10.1111/1460-6984.70292

**Published:** 2026-07-14

**Authors:** Shu En Lee, Genevieve Vuong, Jade Dignam, Annie Hill

**Affiliations:** ^1^ School of Health and Rehabilitation Sciences The University of Queensland Brisbane Queensland Australia; ^2^ Centre of Research Excellence in Aphasia Recovery and Rehabilitation La Trobe University Melbourne Victoria Australia; ^3^ School of Allied Health, Human Services and Sport La Trobe University Melbourne Victoria Australia; ^4^ Queensland Aphasia Research Centre, School of Health and Rehabilitation Sciences The University of Queensland Brisbane Queensland Australia; ^5^ Surgical, Treatment and Rehabilitation Service Education and Research Alliance The University of Queensland and Metro North Hospital and Health Service Brisbane Queensland Australia; ^6^ Faculty of Health Southern Cross University Bilinga Queensland Australia

**Keywords:** aphasia, intensive and comprehensive aphasia program, speech‐language pathologists, telerehabilitation, training

## Abstract

**Background:**

TeleCHAT is the Comprehensive High‐dose Aphasia Treatment program delivered via telerehabilitation. TeleCHAT is a complex intervention, with delivery challenges arising from its comprehensive scope and individualised therapeutic and technological requirements. Thus, training for speech‐language pathologists (SLPs) in the delivery of TeleCHAT is necessary.

**Aims:**

This study aimed to evaluate the acceptability and effectiveness of a training package in equipping SLPs with the knowledge, skills and confidence to deliver TeleCHAT via telerehabilitation.

**Methods and Procedures:**

Three cohorts of SLPs (*n* = 14) completed the TeleCHAT training package, which consisted of theoretical content and practical training. Participants completed a 13‐item satisfaction survey using a 5‐point Likert scale and an observation of technological skills checklist (OTSC) post‐training. Video recordings of four SLPs’ delivery of their first TeleCHAT session was observed by researchers. The SLPs’ independence delivering therapy was rated using the OTSC. Quantitative and qualitative data were analysed using descriptive statistics (mean, standard deviation and range) and content analysis, respectively. Data from each cohort informed improvements to the training package for the next cohort.

**Outcomes and Results:**

Participants self‐rated as moderately to totally independent in the use of telerehabilitation technology post‐training, with high levels of understanding, confidence and preparedness of delivering TeleCHAT. Observations of SLPs’ first TeleCHAT sessions demonstrated total independence with delivering TeleCHAT and troubleshooting technological issues. Content analysis revealed suggestions to improve (a) the content and (b) the structure and delivery of training. The theoretical components transitioned to self‐paced online modules to address the time constraints in SLPs’ workload for Cohorts 2 and 3.

**Conclusion and Implications:**

The training package was well received by participants. Multimodal learning approaches, practical training and specificity of the components of the intensive, comprehensive aphasia program were essential in building confidence in SLPs.

**WHAT THIS STUDY ADDS:**

*What is already known on the subject*

- There is evidence to suggest that formal training equips clinicians with the necessary knowledge and skills for delivering services via telerehabilitation. Existing studies primarily provided content‐based learning but promoted the inclusion of practical and observational training components to build clinician confidence in telerehabilitation.

*What this paper adds to existing knowledge*

- This study highlights the importance of multimodal and specific training, emphasising practical training to build clinician confidence in delivering complex and specialist interventions, such as ICAPs, via telerehabilitation.
- It also provides an example of the design of a clinician training package for telerehabilitation based on behaviour change theory.

*What are the potential or actual clinical implications of this work?*

- The findings of this study advocates for the integration of practical training and content tailored to the specific intervention in telerehabilitation services. By focusing on these aspects in training, clinicians are better prepared to deliver telerehabilitation services, thereby enhancing patient care outcomes.

## 1 Introduction

Aphasia is an acquired language disorder that impairs a person's capacity for language comprehension and expression through speech, writing and/or gestures (Berthier, 2005). Aphasia can have profound impacts on individuals’ functional health recovery (Gialanella et al., 2011), psychosocial wellbeing (Moss et al., 2021), social participation (Davidson et al., 2008), family functioning (Blom Johansson et al., 2022) and overall quality of life (Bullier et al., 2020).

There is Level I evidence (Merlin et al., 2009) supporting the effectiveness of aphasia interventions when delivered at a high dose or intensity (Brady et al., 2022; Brady et al., 2016). Intensive, Comprehensive Aphasia Programs (ICAPs) seek to integrate this evidence with comprehensive therapy approaches that span the World Health Organisation's International Classification of Functioning, Disability and Health (WHO‐ICF) model (Rose et al., 2013, 2022; World Health Organisation [WHO], 2001). The Comprehensive High‐dose Aphasia Treatment program (CHAT) is a modified ICAP, adapted for implementation from the former Aphasia Language Impairment and Functioning Therapy (Aphasia LIFT) program (Rodriguez et al., 2013; Worrall et al., 2024), and has shown positive language gains and lasting positive effects on communication‐related quality of life in persons with aphasia (PWAs) (Dignam et al., 2015; Dignam et al., 2023; Worrall et al., 2024). CHAT provides 50 hours of aphasia therapy, administered over 8 weeks, and includes impairment, activity and participation, and computer‐based interventions, as well as psychosocial group therapy and education (Dignam et al., 2023). However, critical workforce shortages and inequitable geographical distribution of speech‐language services across Australia hinders the delivery of and access to such intensive and comprehensive aphasia treatment (Rose et al., 2014). Other barriers to accessing ICAPs may include clients’ cognitive ability, fatigue, expectations and readiness, transport and mobility issues (Rose et al., 2014; Shrubsole et al., 2019; Monnelly et al., 2023).

Telerehabilitation has shown to be a feasible, effective and equivalent alternative to in‐person treatment across a range of discrete aphasia therapies (Cacciante et al., 2021; Centikaya et al., 2024; Hall et al., 2013), including impairment‐based therapy (Øra et al., 2020), group therapy (Pitt et al., 2019) and functional‐based therapy (Carr et al., 2022). Given the evidence supporting telerehabilitation across various aphasia therapy formats, there was an impetus to translate an intensive, comprehensive aphasia program, such as the CHAT program for delivery via telerehabilitation. A telerehabilitation system configured to deliver CHAT via telerehabilitation, known as TeleCHAT, was developed using a Human Centred Design Framework that considered both users and contextual factors (Vuong et al., 2024). However, the integration of telerehabilitation technology across a comprehensive array of treatment approaches, such as those delivered in TeleCHAT, present unique challenges for the speech‐language pathologists (SLPs) delivering this model of care. Telerehabilitation differs from in‐person rehabilitation in that it demands additional clinical planning and preparation (Thomas et al., 2025), along with the skills to manage telerehabilitation technology effectively (Anil et al., 2023). PWAs also face language‐related barriers in using telerehabilitation and require additional support from SLPs to participate meaningfully (Menger et al., 2020). Therefore, SLPs need specialised training to optimally facilitate PWAs’ participation in TeleCHAT.

The delivery of competent, culturally responsive and evidence‐based telerehabilitation requires a combination of clinical and technical expertise (Anil et al., 2023; Øra et al., 2020; Speech Pathology Australia, 2022). This includes appropriate client selection, operation and maintenance of technology systems, tele‐etiquette and non‐verbal communication skills for the virtual environment (Anil et al., 2023; Lowman et al., 2022; Speech Pathology Australia, 2022). Research has highlighted the importance of clinician training in delivering telerehabilitation for aphasia and stroke. For example, Hilari et al. (2024) identified clinician training in troubleshooting technology and accessing appropriate resources as facilitators for the delivery of aphasia assessment via telerehabilitation. Similarly, a systematic review of the factors influencing stroke telerehabilitation found that clinician training improves the usability and acceptability of telerehabilitation in clinical practice (Stephenson et al., 2022). However, in contexts where formal training opportunities are lacking, SLPs have often relied on self‐guided learning and experimentation to build their telerehabilitation skills (Mohan et al., 2017; Peh et al., 2022). Such limitations in accessing training may have negatively influenced the uptake of telerehabilitation.

The scarcity of formal telerehabilitation training resources aligns with the broader literature gap in training and education in telerehabilitation for health professionals across disciplines. Aimed at understanding the content and delivery methods of telerehabilitation training for clinicians, Edirippulige & Armfield (2017) identified only nine peer‐reviewed articles on formal training programs, while a more recent scoping review by Anil et al. (2025) found just twelve studies across allied health disciplines. Despite the limited literature, both reviews reported positive learning outcomes across diverse training formats. However, neither review identified studies specifically addressing the delivery of aphasia therapy via telerehabilitation. Anil et al. (2025) highlighted the need for further research into evidence‐based curriculum designs for telerehabilitation training. Nevertheless, the findings offer foundational evidence to inform the development of a training package that equips SLPs with the knowledge and skills to deliver TeleCHAT.

Practical and observational learning were highly recommended in telerehabilitation training for clinicians to build skills and confidence (Edirippulige & Armfield, 2017). For example, a practical training component enhanced an online telerehabilitation training program, by providing its participants with essential opportunities to use different telerehabilitation technologies (Edirippulige et al., 2012). A telerehabilitation training program for post‐acute outpatient clinicians also identified simulation training, involving roleplay and troubleshooting technological issues, as a facilitator for improving clinician skills and confidence in delivering telerehabilitation services (Morton et al., 2023). Additionally, Nalder et al. (2018) found that combining intervention manuals, telerehabilitation technology training, and continuous technical and clinical support were helpful in ensuring adherence to treatment protocols when translating in‐person interventions to telerehabilitation.

Telerehabilitation competency is an explicit skill set that needs to be directly targeted through training (Anil et al., 2025; Anil et al., 2023). However, no studies to date have evaluated training specifically for SLPs to deliver comprehensive aphasia therapy programs via telerehabilitation. To address this gap, this research team developed a comprehensive training package for the telerehabilitation delivery of an intensive comprehensive aphasia program (TeleCHAT). This study aims to evaluate the acceptability and effectiveness of the TeleCHAT training package in equipping SLPs with the knowledge, skills and confidence to deliver TeleCHAT.

## 2 Methods

### 2.1 Study Design

This mixed methods prospective cohort study evaluated the effectiveness and acceptability of a comprehensive telerehabilitation training package developed to support the delivery of TeleCHAT. Acceptability was defined as SLPs’ satisfaction and perceptions of the training program. This study was conducted as part of a broader program of research to evaluate the feasibility, usability, acceptability and efficacy of TeleCHAT. The Mixed Methods Reporting in Rehabilitation & Health Sciences (MMR‐RHS) checklist (Tovin & Wormley, 2023) was followed to ensure systematic reporting.

### 2.2 Ethical Clearance

Ethics approval was received from the relevant Human Research Ethics Committees and all participants provided written informed consent prior to participation. This study took place in 2021–2023.

### 2.3 Participants

A total of 14 SLPs were recruited through the Surgical Treatment and Rehabilitation Service (STARS) Education and Research Alliance, The University of Queensland and Metro North Health, Queensland, Australia. All participants were certified practising SLPs with a minimum qualification of Bachelor of Speech Pathology or equivalent, and previous experience of delivering aphasia therapy. Four SLPs delivered TeleCHAT to PWAs within the broader TeleCHAT research project. The remaining participants consented to this study as part of their professional development. Participation was voluntary and was not a requirement of their employment in the health, hospital or research service. The participants engaged in training across three cohorts of TeleCHAT.

### 2.4 Development and Delivery of TeleCHAT Training Package

The TeleCHAT training package was developed by the research team consisting of a doctoral student and three experienced telerehabilitation and aphasia research SLPs. The training package was developed with the aim to increase SLP's knowledge, skills and confidence in delivering TeleCHAT, and was informed by behaviour change theory (Cane et al., 2012; Michie et al., 2005). Feedback from each cohort was integrated into the ongoing improvement of the training package.

Each participant engaged in an average of five hours of TeleCHAT training. Participants first completed two to three hours of theoretical content learning (see Table 1 for learning topics). This theoretical content was initially presented by a researcher in‐person. Participants were also provided with a comprehensive, resource package including the TeleCHAT training manual, digital therapy templates, checklists, and task analysis sheets. All resources were stored and accessed through secured cloud drives—CloudStor 2023 AARNET and the University of Queensland Research Data Manager.

**TABLE 1:** Learning topics in TeleCHAT online training modules.

Online modules	Learning topics
1	Background—Evidence for telerehabilitation and TeleCHAT
2	Overview of the TeleCHAT program
3	Task analysis of therapy activities to be implemented via telerehabilitation
4	TeleCHAT technology—Design solutions
5	Training people with aphasia for tele‐assessment and telerehabilitation
6	TeleCHAT usual therapy session, rapport building tips and therapy provision considerations
7	Management of technological issues and adverse events
8	Practical therapy session preparation

Following the theoretical content sessions, the participants engaged in a two‐hour practical training session as a group. This session was an opportunity for participants to perform practical tasks such as, setting up Zoom on their devices. They also roleplayed as both the SLPs and recipient of therapy, to experience the delivery of typical therapy activities for impairment, functional, group and computer therapy in simulated telerehabilitation sessions. Each participant was required to have two devices (e.g., a laptop and iPad or desktop and iPad) and preferably used the device they would use for delivering therapy. Participants accessed the training either from their office within Queensland Health or the telerehabilitation suites at Queensland Aphasia Research Centre (QARC). While it was also recommended that participants have at least two screens, this was not feasible for some Queensland Health SLPs in their usual workspace.

After completing the group practical training, the four participants who delivered TeleCHAT were offered additional one‐on‐one training via Zoom from the research team, where they were allowed more time to practice troubleshooting technological issues or delivering specific therapy tasks for TeleCHAT.

### 2.5 Data Collection

#### 2.5.1 Demographic Survey

Prior to training, the participants completed a survey to provide demographic information and details of their previous experiences of telerehabilitation, use of videoconferencing and engagement in training workshops related to telerehabilitation (see Supplemental 1).

#### 2.5.2 Observation of Technological Skills Checklist

An observation of technological skills checklist (OTSC) adapted from Hill & Breslin (2016) (see Supplemental 2) was used to assess the participants’ independence in using the technological devices and videoconferencing software to conduct a telerehabilitation therapy session immediately post‐training. Participants rated their own skills on a three‐point Likert scale (1 = Not at all independent, 2 = Moderately independent, 3 = Totally independent), and where applicable, indicated whether they required assistance from a researcher or the training manual to complete each task. The OTSC was distributed via email and then stored securely in CloudStor 2023 AARNET and RDM.

#### 2.5.3 Effectiveness of Telerehabilitation Training

The first author conducted a full review of the recordings of the first therapy sessions delivered by the four participants who provided TeleCHAT therapy. The OTSC was used to assess their independence during these sessions. In addition, the participants documented any errors encountered during therapy delivery using a self‐reported technology error log. The error logs from their first therapy sessions were extracted into a Microsoft Excel spreadsheet and compared with observations of the session recordings.

#### 2.5.4 Satisfaction Survey

A 13‐item survey was purposefully designed by the research team to elicit qualitative and quantitative data regarding participant satisfaction with the training (see Supplemental 3). The satisfaction survey was completed the day of or the day after training.

The survey consisted of 13 questions using a five‐point Likert scale (1 = strongly disagree to 5 = strongly agree) to assess the participants’ knowledge, confidence and preparedness after training, as well as their perceptions of how well the training package prepared them to deliver TeleCHAT and resolve potential technological issues. Two open‐ended response boxes were available for participants to provide feedback on possible areas of improvement for the training package. Survey responses were kept anonymous using a unique code for each participant and collected via online survey software, Qualtrics, or printed paper copies. Paper copies were scanned and stored securely on RDM.

#### 2.5.5 Observational Feedback on Content and Structure

The senior author observed the in‐person practical sessions and provided field notes to suggest improvements for the training package on content and structure. The senior author has over 20 years of experience in telerehabilitation development and evaluation.

### 2.6 Data Analysis

Quantitative data from the demographic survey, OTSC and satisfaction survey were analysed descriptively using Microsoft Excel. Open‐ended responses from the satisfaction survey and the observation field notes were uploaded to Microsoft Excel and analysed using inductive content analysis (Graneheim & Lundman, 2004). Field notes and open‐ended responses were coded into meaning units by the first author. The meaning units were then condensed into descriptions close to the text and, where possible, into an interpretation of their underlying meaning to allow for the identification of common themes. This was then cross‐checked by the remaining three authors. Any discrepancies were discussed within the team until consensus was reached to enhance reliability of the outcomes. The first author was not involved in the development and delivery of the training, to reduce potential bias and enhance the credibility of the analysis.

During the delivery of the training, participant responses were reviewed in an ongoing, informal manner to support iterative refinements to the training process. This review did not involve a structured or systematic analysis approach, and no formal interim analysis was conducted. Formal qualitative analyses were conducted after the completion of data collection and were performed on the full dataset across all cohorts.

## 3 Results

### 3.1 Participants

All 14 participants completed the TeleCHAT training program. Their demographic details are provided in Table 2. On average, the participants (*n* = 12) had approximately six years of experience delivering aphasia therapy (range = 0.5–25 years). Before undergoing TeleCHAT training, 50% (*n* = 7) of the participants had previous experience in delivering aphasia therapy via telerehabilitation. Additionally, 50% (*n* = 7) of the participants had previously participated in professional development related to telerehabilitation. The reported professional development activities included online learning modules and webinars, informal training delivered by workplaces, seminars, special interest groups, and reading journal articles.

**TABLE 2:** Participant demographic information (*n* = 14).

Parameter	*n*
Gender	
Female	14
Male	0
Highest level of education completed	
Bachelor	2
Bachelor with Honours	8
Master	3
PhD	1
Years of experience delivering aphasia rehabilitation	(*n* = 12)
<1	4
1–5	4
6–10	1
11–15	1
16–20	1
21–25	1
Previous experience of delivering aphasia therapy via telerehabilitation	
Yes	7
No	7
Previous experience of upskilling or professional development related to telerehabilitation	
Yes	7
No	7

### 3.2 Independence in Using the Technology for Telerehabilitation

Twelve participants completed the OTSC immediately after the group training; two participants did not complete this component. The results of the OTSC are reported in Table 3. Overall, participants’ self‐reported independence levels in using the telerehabilitation technology immediately post‐training were varied across domains, but all fell within moderately independent to totally independent (range = 2–3). Although all participants rated their independence in setting up videoconferencing software at the highest score (Mdn = 3), one participant did refer to the training manual, and two participants required direct support in this domain. It is also notable that no participants required any support with setting up and using the computer (Mdn = 3). Greatest support was indicated for ‘within therapy activities’, where four participants used the training manual, while three participants received direct support.

**TABLE 3:** Frequency of participants’ self‐reported independence in technological skills immediately post‐training (*n* = 12).

Domains	Ratings^*^				Support required	
	1	2	3	Median	Referred to training manual	Required direct support
Set up and use computer (*n* = 12)	0	1	11	3	0	0
Set up and use iPad/tablet (*n* = 9)	0	0	9	3	0	1
Therapy preparation (*n* = 11)	0	3	8	3	2	0
Set up videoconferencing software (*n* = 12)	0	0	12	3	1	2
Within therapy activities (*n* = 12)	0	2	10	3	4	3
Finish therapy session (*n* = 11)	0	4	7	3	1	1

*Note*: *n* values vary by item due to incomplete responses; denominators are shown for each item.

^*^Domain ratings reflect the median of subdomain ratings per participant, reported on a 3‐point scale (1 = Not at all independent, 2 = Moderately independent, 3 = Totally independent).

Objective evaluation of the four SLPs’ first TeleCHAT therapy sessions demonstrated total independence (Mdn = 3) across all domains of the OTSC. These first sessions included one computer therapy, one functional therapy and two impairment therapy sessions. Across these sessions, a total of five technological errors were recorded. When observing the session recordings, the participants exhibited full independence in troubleshooting these five technological errors and showed flexibility in problem solving.

### 3.3 Satisfaction Towards the Training Package

Upon completion of training, all 14 participants responded to the satisfaction survey and reported high satisfaction scores across all survey items (Mdn = 5, range: 3–5; see Table 4). The high ratings in knowledge, confidence and preparedness suggest that the participants perceived the training package as helpful in preparing SLPs to deliver TeleCHAT. Moreover, 50% (*n* = 7) of the participants explicitly expressed positive views in the open‐ended responses, stating that the training package comprehensively addressed the skills needed to deliver TeleCHAT.

**TABLE 4:** Frequency of participants’ responses on the satisfaction survey (*n* = 14).

Items	Ratings^*^					Median	Range
	1	2	3	4	5		
The TeleCHAT program has been explained to me. (*n* = 13)	0	0	0	0	13	5	5–5
I understand all the components of TeleCHAT. (*n* = 12)	0	0	0	0	12	5	5–5
I understand what is required of me to deliver TeleCHAT. (*n* = 12)	0	0	0	0	12	5	5–5
The simulation session helped me understand how to use the computer and videoconferencing software (telerehabilitation system). (*n* = 14)	0	0	0	1	13	5	4–5
I am confident using the telerehabilitation system. (*n* = 14)	0	0	1	9	4	4	3–5
The mock technical difficulty procedure helped me understand how to troubleshoot any technical difficulties experienced. (*n* = 14)	0	0	0	6	8	5	4–5
I am confident that I can troubleshoot any technical difficulties experienced from my end. (*n* = 14)	0	0	0	12	2	4	4–5
I am confident that I can help the PWA troubleshoot any technical difficulties they may have on their end. (*n* = 14)	0	0	3	10	1	4	3–5
I know that I can refer to the TeleCHAT manual to help me resolve technical difficulties. (*n* = 14)	0	0	0	4	10	5	4–5
I know the chain of contacts to refer any technological issues to. (*n* = 13)	0	0	0	5	8	5	4–5
I am confident that I can deliver all the components of TeleCHAT over telerehabilitation. (*n* = 12)	0	0	0	10	2	4	4–5
There was a suitable mix of presentation and practical sessions. (*n* = 14)	0	0	0	5	9	5	4–5
The training session has prepared me well for delivering TeleCHAT. (*n* = 13)	0	0	0	5	8	5	4–5

*Note*: *n* values vary by item due to incomplete responses; denominators are shown for each item.

^*^Rating on 5‐point scale with 1 = strongly disagree, 2 = disagree, 3 = neither agree nor disagree, 4 = agree, 5 = strongly agree.

Notably, items associated with increased knowledge generally had a higher mean score than items associated with confidence. All participants reported a maximum score of 5 regarding their knowledge of the TeleCHAT program, its components and what is required of the participants to deliver TeleCHAT (Items 1–3). In contrast, confidence in helping PWAs with troubleshooting technological difficulties had the highest frequencies of ratings of 3 (*n = 3*) and 4 (*n* = 10), with a median of 4 on the 5‐point Likert scale. Additionally, while the simulation session was rated highly for improving understanding of the telerehabilitation system (Mdn = 5; range = 4–5), the ratings were slightly lower for improving confidence in using the telerehabilitation system (Mdn = 4; range = 3–5).

### 3.4 Improving the Telechat Training Package

Content analysis of open‐ended survey responses and observation fieldnotes revealed that the suggestions for the TeleCHAT training package corresponded to two main categories, the content of training, and the structure and delivery of training. Responses from the observing researcher and participants are described in Table 5.

**TABLE 5:** Condensation and interpretation of feedback and suggestions on the TeleCHAT training package.

Category: Content of Training		
Subcategory	Participants	Observing researcher
Research evidence	No feedback or suggestions reported	Evidence on the specifics and importance of the components of training. Evidence for telerehabilitation in specific clinical areas. Evidence acknowledges that telerehabilitation can be exhausting for SLPS.
Background knowledge and overview of training	No feedback or suggestions reported	Combine background knowledge about TeleCHAT to the clinical context (telerehabilitation). Provide widely known examples of telerehabilitation outside of speech‐language pathology. Provide context on the research aim for TeleCHAT.
TeleCHAT technology and setup	No feedback or suggestions reported	Provide a comparison table of telerehabilitation software or equipment in terms of functionality, efficiency and capability to perform tasks, including videoconferencing. Discuss clinical feasibility of using Streamdeck, camera view and a stylus. Ensure devices are fully charged during therapy period. Integrate setup checklists and session resources into the context of the feasibility of TeleCHAT. Include information on audio setup for clients. Revisit ways of contacting clients before therapy session.
TeleCHAT delivery	Include more practical examples of task analysis and note‐taking during therapy. Resources and ‘cheat sheets’ included in the training package are comprehensive and practical for therapy implementation.	Task analysis and choice of technology enable participation from PWA of all degrees of severity. Rapport building is an essential skill for telerehabilitation that SLPs can learn and develop. Provide tips and ‘cheat sheet’ on rapport building. Define the goals of the practical training session.

Category: Structure and Delivery of Training		
Sub‐Category	Participants	Observing researcher
Practical sessions	Conduct separate practical sessions for demonstration of group therapy and troubleshooting on the clients’ end. Recruit past participants to support training. Include site‐specific practical tasks, such as delivery of therapy using site‐specific virtual platforms.	Record practical sessions for use in future training. Conduct separate sessions for demonstration of technology and practical training. Discuss setup checklists in the tele‐suites at QARC to provide clinical context. Having two SLPs per session is ideal but may not be clinically feasible.
Theoretical sessions	Fix the popping of audio in video resources. Provide at least 1–2 weeks to complete online‐training modules.	Start presentation slides with an overview of training to orient participants to the training session. Start task analysis example with therapy tasks to provide clinical context.
Pre‐training resources and home practice	Distribute instructions of therapy tasks before practical training session.	Provide case study in pre‐training resources or at the end of training for home practice. Distribute instructions of setup checklists and session resources before practical training session.

#### 3.4.1 Content of Training

The observing researcher provided several suggestions for refining the evidence section of the training package, for example, ‘In the evidence section—just temper the scope of evidence for telerehab [telerehabilitation]—there is evidence but it's in specific areas, not across all of the areas listed [on the slides]’. Furthermore, she recommended acknowledging the difficulty of implementing aphasia therapy through telerehabilitation, ‘you could acknowledge that delivering therapy via telerehabilitation is more challenging for SLPs and tiring’. These suggestions were iteratively incorporated into the training package as they arose. Additionally, participants requested the inclusion of practical examples such as ‘Maybe some more practical application of task analysis for therapy tasks’ (SLP1) and ‘some practical examples around note tking [taking] during Tx [therapy] while managing the tech [technology]’ (SLP2).

#### 3.4.2 Structure and Delivery of Training

Both the participants and the observing researcher reported that more practical training sessions for specific tasks was required. This was illustrated by quotes such as, ‘*A separate mock session focussing on troubleshooting from the PWA's end would be helpful*’. (SLP1) and ‘*Probably need 2 hands‐on training sessions—one where you demo [demonstrate] the tech and one that is actually hands‐on and letting everyone have a go*’. (Observing researcher). Preparation of training resources were also recommended to be supplied prior to the practical training session. For example, one participant stated, ‘*Maybe, a pre‐meeting email with a standard selection of therapy tasks to trial, with instructions of tasks to implement during the training to reduce training time?*’ (SLP13). In terms of the timeframe between the theoretical and practical components, SLP15 suggested for the research team to provide ‘*More time/notice to complete the pre‐practical training components (e.g., 1–2 weeks)*’. Therefore, to address participants’ time constraints and the scheduling difficulties of in‐person sessions, the research team transitioned the theoretical component into self‐paced online modules for Cohorts 2 and 3. Due to COVID‐19 restrictions, the practical training sessions for Cohort 2 were conducted entirely via Zoom. For Cohort 3, these sessions resumed to being delivered in‐person.

## 4 Discussion

This study demonstrated that a comprehensive, multimodal training package to support the delivery of an ICAP via telerehabilitation (TeleCHAT) was effective and was highly satisfactory to SLP participants. SLPs reported high levels of knowledge, confidence and preparedness to deliver TeleCHAT after undertaking the training program. While post‐training independence in using telerehabilitation technology varied across the different domains of technological skills, all participants reported moderate to high levels of independence. These self‐reports were supported by objective observations that showed that SLPs were able to deliver their first TeleCHAT sessions independently.

The effectiveness of the training was reflected in the participants’ high perceived gains in knowledge, skills and confidence to deliver TeleCHAT, as well as their observed independence in delivering TeleCHAT. Objective assessment of the four participants who delivered TeleCHAT found that they were fully independent, not only in conducting TeleCHAT sessions but also in troubleshooting technological issues during their initial sessions. These successful first‐time TeleCHAT sessions underscore the training's impact in equipping SLPs with the knowledge and practical skills required for effective clinical application. However, participants reported lower independence and required more support when applying technological skills for therapy preparation and delivery, particularly for troubleshooting technological issues. These data highlight the need for explicit training in managing the nuanced technological functionalities for telerehabilitation. Similarly, Erickson et al. (2022) found that experienced SLPs required both clinical and technical support when transitioning the Lidcombe fluency program to telerehabilitation, despite prior proficiency in delivering the same program in‐person. Moreover, during TeleCHAT, SLPs had to take on responsibilities of managing technology to enable the participation of PWAs whose language deficits may have limited their ability to independently navigate telerehabilitation technology (Menger et al., 2020). This dual requirement for clinical and technical skills highlights the importance of integrating simulations, technology‐focused training and troubleshooting into our training package to ensure comprehensive preparation for telerehabilitation delivery of a complex intervention to people with aphasia.

Overall, the satisfaction survey yielded positive feedback, indicating that participants viewed the training package favourably for preparing them in the delivery of TeleCHAT. A key strength of the TeleCHAT training package was its multimodal learning approach that combined a theoretical component delivered through self‐paced online modules with synchronous practical training sessions. This design was structured to target various domains of behaviour change, as outlined in implementation science frameworks (Cane et al., 2012; Michie et al., 2005). The theoretical component comprehensively addressed ‘knowledge’ acquisition, as evidenced by consistently high survey ratings for items related to knowledge and understanding, while practical training supported ‘skills’ acquisition and potentially enhanced SLP confidence (i.e., ‘beliefs about capabilities’) to deliver an ICAP via telerehabilitation. Furthermore, the flexibility of the training delivery, accommodating both in‐person and online (Zoom) sessions likely contributed to participant satisfaction. The inclusion of therapy resources and manuals also supported ‘memory, attention and decision processes’. Checklists and templates facilitated the practical delivery of therapy activities, while the training manual and troubleshooting guides provided ongoing support for translating in‐person therapy to telerehabilitation, as reported in a previous study (Nalder et al., 2018). Using behaviour change theory to construct the TeleCHAT training package presents an innovative approach, as existing telerehabilitation training literature rarely incorporates explicit theoretical frameworks in their curriculum design (Anil et al., 2025). Grounding the training in behaviour change theory enhances its clinical applicability and aligns with implementation research that highlighted clinician training as a key facilitator for effective aphasia practices (Shrubsole et al., 2023).

Regarding areas for improvement, the comparatively lower ratings in participants’ confidence levels highlight a need for more practical training to translate theoretical knowledge into confident clinical application. Participants’ requests for increased skill‐based training further reinforces this need. Previous research supports the importance of practical training, noting that clinicians require increased time to develop experience in delivering telerehabilitation and build familiarity with telerehabilitation technology (Caughlin et al., 2020; Edirippulige & Armfield, 2017). The request for more practical training may also stem from the complexity of TeleCHAT being an ICAP that involves multiple therapy approaches tailored to each PWA (Monnelly et al., 2023). Thus, an important consideration of this training package was to allow for the transfer of skills across different therapy tasks. Our training package covered four broad aphasia therapy approaches (i.e., impairment, activity and participation, group and computer therapy), each of which included multiple therapy tasks that could be personalised to the PWA. Practical training thereby becomes essential for familiarising SLPs with the telerehabilitation technology and for building their skills in delivering these specific therapy tasks effectively.

### 4.1 Limitations and Future Directions

Only four participants completed the training package with the intention of delivering TeleCHAT immediately post‐training. Consequently, the remaining 10 participants may not have provided specific feedback with the intent of delivering TeleCHAT; instead, their feedback could have been more reflective of their general clinical contexts or applicable to treatments other than ICAPs. This divergence in intentions may have influenced the relevance and specificity of their feedback regarding the training package. Another limitation of this study was the use of a satisfaction survey and OTSC that were not formally validated. However, both tools were developed by experts in telerehabilitation and aphasia rehabilitation, supporting their content validity and practical relevance.

Future studies could explore other variables that influence skill acquisition, such as the timing of training delivery. For example, Erickson et al. (2022) observed that SLPs had difficulties recalling the content and skills of telerehabilitation when training was provided three‐months in advance. Likewise, as practical training emerged as a crucial component for the consolidation of skills in the present study, future investigations should formally evaluate the optimal amount, specificity and timing of these sessions to determine what is both feasible and effective for long‐term skill retention.

## 5 Conclusion

The training package developed in this study was acceptable to 14 SLPs and showed effectiveness in equipping four SLPs with the skills to deliver TeleCHAT. The study highlighted the importance of explicit and tailored training packages for the delivery of specialist and complex interventions, such as ICAPs. SLPs required both theoretical knowledge and practical skills to confidently and independently deliver telerehabilitation services; hence, this study suggests that multimodality training with an emphasis on practical training can enhance clinical and technical skills.

## Ethics Statement

Ethics approval was received from the relevant Human Research Ethics Committees (HREC/2020/QRBW/61636 and 2020/HE002118).

## Conflicts of Interest

The authors declare no conflicts of interest.

## Supporting information



Supporting Information: jlcd70292‐supp‐0001‐SuppMat.docx

Supporting Information: jlcd70292‐supp‐0002‐SuppMat.docx

Supporting Information: jlcd70292‐supp‐0003‐SuppMat.docx

## Data Availability

The data that support the findings of this study are available on request from the corresponding author. The data are not publicly available due to privacy or ethical restrictions.

## References

[jlcd70292-bib-0001] Anil, K. , A. Bird , K. Bridgman , et al. 2025. “Telehealth Training and Education for Allied Health Professionals: A Scoping Review.” Telemedicine Reports 6, no. 1: 76–90. 10.1089/tmr.2024.0083. 40308562 PMC12040532

[jlcd70292-bib-0002] Anil, K. , A. R. Bird , K. Bridgman , et al. 2023. “Telehealth Competencies for Allied Health Professionals: A Scoping Review.” Journal of Telemedicine and Telecare 31, no. 4: 487–499. 10.1177/1357633x231201877. 37787172 PMC12044212

[jlcd70292-bib-0003] Berthier, M. L. 2005. “Poststroke Aphasia.” Drugs & Aging 22, no. 2: 163–182. 10.2165/00002512-200522020-00006. 15733022

[jlcd70292-bib-0004] Blom Johansson, M. , M. Carlsson , P. Östberg , and K. Sonnander . 2022. “Self‐Reported Changes in Everyday Life and Health of Significant Others of People With Aphasia: A Quantitative Approach.” Aphasiology 36, no. 1: 76–94. 10.1080/02687038.2020.1852166.

[jlcd70292-bib-0005] Brady, M. C. , M. Ali , K. VandenBerg , et al. 2022. “Dosage, Intensity, and Frequency of Language Therapy for Aphasia: A Systematic Review‐Based, Individual Participant Data Network Meta‐Analysis.” Stroke; A Journal of Cerebral Circulation 53: 956–967. 10.1161/STROKEAHA.121.035216. PMC8884127 34847708

[jlcd70292-bib-0006] Brady, M. C. , H. Kelly , J. Godwin , P. Enderby , and P. Campbell . 2016. “Speech and Language Therapy for Aphasia Following Stroke.” Cochrane Database of Systematic Reviews 2016, no. 6: CD000425. 10.1002/14651858.CD000425.pub4. 27245310 PMC8078645

[jlcd70292-bib-0007] Bullier, B. , H. Cassoudesalle , M. Villain , et al. 2020. “New Factors That Affect Quality of Life in Patients With Aphasia.” Annals of Physical and Rehabilitation Medicine 63, no. 1: 33–37. 10.1016/j.rehab.2019.06.015. 31352062

[jlcd70292-bib-0008] Cacciante, L. , P. Kiper , M. Garzon , et al. 2021. “Telerehabilitation for People With Aphasia: A Systematic Review and Meta‐Analysis.” Journal of Communication Disorders 92: 106111. 10.1016/j.jcomdis.2021.106111. 34052617

[jlcd70292-bib-0009] Cane, J. , D. O'Connor , and S. Michie . 2012. “Validation of the Theoretical Domains Framework for use in Behaviour Change and Implementation Research.” Implementation Science 7, no. 1: 37–37. 10.1186/1748-5908-7-37. 22530986 PMC3483008

[jlcd70292-bib-0010] Carr, P. , D. Moser , S. Williamson , G. Robinson , and S. Kintz . 2022. “Improving Functional Communication Outcomes in Post‐Stroke Aphasia via Telepractice: An Alternative Service Delivery Model for Underserved Populations.” International Journal of Telerehabilitation 14, no. 2: e6531. 10.5195/ijt.2022.6531. 38026567 PMC10681046

[jlcd70292-bib-0011] Caughlin, S. , S. Mehta , H. Corriveau , et al. 2020. “Implementing Telerehabilitation After Stroke: Lessons Learned From Canadian Trials.” Telemedicine and e‐Health 26, no. 6: 710–719. 10.1089/tmj.2019.0097. 31633454

[jlcd70292-bib-0012] Centinkaya, B. , K. Twomey , B. Bullard , S. El Kouaissi , and P. Conroy . 2024. “Telerehabilitation of Aphasia: A Systematic Review of the Literature.” Aphasiology 38, no. 7: 1271–1302. 10.1080/02687038.2023.2274621.

[jlcd70292-bib-0013] Davidson, B. , T. Howe , L. Worrall , L. Hickson , and L. Togher . 2008. “Social Participation for Older People With Aphasia: The Impact of Communication Disability on Friendships.” Topics in Stroke Rehabilitation 15, no. 4: 325–340. 10.1310/tsr1504-325. 18782736

[jlcd70292-bib-0014] Dignam, J. , D. Copland , E. McKinnon , et al. 2015. “Intensive Versus Distributed Aphasia Therapy.” Stroke; A Journal of Cerebral Circulation 46, no. 8: 2206–2211. 10.1161/STROKEAHA.115.009522. 26106114

[jlcd70292-bib-0015] Dignam, J. K. , K. Shrubsole , K. O'Brien , et al. 2023. “Implementation of the Comprehensive High‐Dose Aphasia Treatment (CHAT) Program: “A Different way of Operating”.” Aphasiology 39: 64–92. 10.1080/02687038.2023.2287766.

[jlcd70292-bib-0016] Edirippulige, S. , and N. R. Armfield . 2017. “Education and Training to Support the use of Clinical Telehealth: A Review of the Literature.” Journal of Telemedicine and Telecare 23, no. 2: 273–282. 10.1177/1357633x16632968. 26892005

[jlcd70292-bib-0017] Edirippulige, S. , A. C. Smith , N. R. Armfield , M. Bensink , and R. Wootton . 2012. “Student Perceptions of a Hands‐On Practicum to Supplement an Online eHealth Course.” Journal of Medical Internet Research 14, no. 6: e182. 10.2196/jmir.2029. 23246840 PMC3799484

[jlcd70292-bib-0018] Erickson, S. , K. Bridgman , L. Furlong , and H. Stark . 2022. “Speech‐Language Pathologist Perspectives of the Implementation of Telepractice‐Delivered Stuttering Treatment for School‐Age Children.” Language, Speech, and Hearing Services in Schools 53, no. 1: 30–43. 10.1044/2021_LSHSS-20-00167. 34752153

[jlcd70292-bib-0019] Gialanella, B. , M. Bertolinelli , M. Lissi , and P. Prometti . 2011. “Predicting Outcome After Stroke: The Role of Aphasia.” Disability and Rehabilitation 33, no. 2: 122–129. 10.3109/09638288.2010.488712. 20521995

[jlcd70292-bib-0020] Graneheim, U. H. , and B. Lundman . 2004. “Qualitative Content Analysis in Nursing Research: Concepts, Procedures and Measures to Achieve Trustworthiness.” Nurse Education Today 24, no. 2: 105–112. 10.1016/j.nedt.2003.10.001. 14769454

[jlcd70292-bib-0021] Hall, N. , M. Boisvert , and R. Steele . 2013. “Telerehabilitation in the Assessment and Treatment of Individuals With Aphasia: A Systematic Review.” International Journal of Telerehabilitation 5, no. 1: 27–38. 10.5195/ijt.2013.6119. 25945211 PMC4296832

[jlcd70292-bib-0022] Hilari, K. , A. Roper , S. Northcott , and N. Behn . 2024. “Telehealth Practice in Aphasia: A Survey of UK Speech and Language Therapists, With a Focus on Assessment.” International Journal of Language & Communication Disorders 59, no. 4: 1296–1307. 10.1111/1460-6984.12996. 38156767

[jlcd70292-bib-0023] Hill, A. J. , and H. M. Breslin . 2016. “Refining an Asynchronous Telerehabilitation Platform for Speech‐Language Pathology: Engaging End‐Users in the Process.” Frontiers in Human Neuroscience 10: 640. 10.3389/fnhum.2016.00640. 28066211 PMC5168427

[jlcd70292-bib-0024] Lowman, J. , J. Walker , and K. T. Houston . 2022. “Preparing Speech–Language Pathology Graduate Students for Effective Telepractice.” Topics in Language Disorders 42, no. 2: 107–126. 10.1097/TLD.0000000000000279.

[jlcd70292-bib-0025] Menger, F. , J. Morris , and C. Salis . 2020. “The Impact of Aphasia on Internet and Technology use.” Disability and Rehabilitation 42, no. 21: 2986–2996. 10.1080/09638288.2019.1580320. 30982360

[jlcd70292-bib-0026] Merlin, T. , A. Weston , and R. Tooher . 2009. “Extending an Evidence Hierarchy to Include Topics Other Than Treatment: Revising the Australian ‘Levels of Evidence’.” BMC Medical Research Methodology 9: 34. 10.1186/1471-2288-9-34. 19519887 PMC2700132

[jlcd70292-bib-0027] Michie, S. 2005. “Making Psychological Theory Useful for Implementing Evidence Based Practice: A Consensus Approach.” Quality and Safety in Health Care 14, no. 1: 26–33. 10.1136/qshc.2004.011155. 15692000 PMC1743963

[jlcd70292-bib-0028] Mohan, H. S. , A. Anjum , and P. K. S. Rao . 2017. “A Survey of Telepractice in Speech‐Language Pathology and Audiology in India.” International Journal of Telerehabilitation 9, no. 2: 69–80. 10.5195/ijt.2017.6233. 29238451 PMC5716619

[jlcd70292-bib-0029] Monnelly, K. , J. Marshall , L. Dipper , and M. Cruice . 2023. “Intensive and Comprehensive Aphasia Therapy—A Survey of the Definitions, Practices and Views of Speech and Language Therapists in the United Kingdom.” International Journal of Language & Communication Disorders 58, no. 6: 2077–2102. 10.1111/1460-6984.12918. 37394906

[jlcd70292-bib-0030] Morton, D. , S. Fulton , M. Harris , et al. 2023. “Development and Implementation of a Training Package to Improve the Confidence, Skills and Knowledge of Multi‐Disciplinary Clinicians in the use of Telerehabilitation for Outpatient Services.” Journal of Adult and Continuing Education 29: 147797142211303. 10.1177/14779714221130376.

[jlcd70292-bib-0031] Moss, B. , S. Northcott , N. Behn , et al. 2021. “‘Emotion is of the Essence. … Number One Priority’: A Nested Qualitative Study Exploring Psychosocial Adjustment to Stroke and Aphasia.” International Journal of Language & Communication Disorders 56, no. 3: 594–608. 10.1111/1460-6984.12616. 33826205

[jlcd70292-bib-0032] Nalder, E. , E. Marziali , D. R. Dawson , and K. Murphy . 2018. “Delivering Cognitive Behavioural Interventions in an Internet‐Based Healthcare Delivery Environment.” British Journal of Occupational Therapy 81, no. 10: 591–600. 10.1177/0308022618760786.

[jlcd70292-bib-0033] Øra, H. P. , M. Kirmess , M. C. Brady , H. Sørli , and F. Becker . 2020. “Technical Features, Feasibility, and Acceptability of Augmented Telerehabilitation in Post‐Stroke Aphasia—Experiences From a Randomized Controlled Trial.” Frontiers in Neurology 11: 671–671. 10.3389/fneur.2020.00671. 32849176 PMC7411384

[jlcd70292-bib-0034] Peh, H. P. , K. Yee , and E. J. N. Mantaring . 2022. “Changes in Telepractice use and Perspectives Among Speech and Language Therapists in Singapore Through the COVID‐19 Pandemic.” International Journal of Language & Communication Disorders 58, no. 3: 802–812. 10.1111/1460-6984.12823. 36412089

[jlcd70292-bib-0035] Pitt, R. , D. Theodoros , A. J. Hill , and T. Russell . 2019. “The Impact of the Telerehabilitation Group Aphasia Intervention and Networking Programme on Communication, Participation, and Quality of Life in People With Aphasia.” International Journal of Speech‐Language Pathology 21, no. 5: 513–523. 10.1080/17549507.2018.1488990. 30200788

[jlcd70292-bib-0036] Rodriguez, A. D. , L. Worrall , K. Brown , et al. 2013. “Aphasia LIFT: Exploratory Investigation of an Intensive Comprehensive Aphasia Programme.” Aphasiology 27, no. 11: 1339–1361. 10.1080/02687038.2013.825759.

[jlcd70292-bib-0037] Rose, M. L. , L. R. Cherney , and L. E. Worrall . 2013. “Intensive Comprehensive Aphasia Programs: An International Survey of Practice.” Topics in Stroke Rehabilitation 20, no. 5: 379–387. 10.1310/tsr2005-379. 24091280

[jlcd70292-bib-0038] Rose, M. L. , A. Ferguson , E. Power , L. Togher , and L. Worrall . 2014. “Aphasia Rehabilitation in Australia: Current Practices, Challenges and Future Directions.” International Journal of Speech‐Language Pathology 16, no. 2: 169–180. 10.3109/17549507.2013.794474. 23777446

[jlcd70292-bib-0039] Rose, M. L. , J. E. Pierce , V. L. Scharp , et al. 2022. “Developments in the Application of Intensive Comprehensive Aphasia Programs: An International Survey of Practice.” Disability and Rehabilitation 44, no. 20: 5863–5877. 10.1080/09638288.2021.1948621. 34251946

[jlcd70292-bib-0040] Shrubsole, K. , D. Copland , A. Hill , et al. 2023. “Development of a Tailored Intervention to Implement an Intensive and Comprehensive Aphasia Program (ICAP) Into Australian Health Services.” Aphasiology 37, no. 9: 1386–1409. 10.1080/02687038.2022.2095608.

[jlcd70292-bib-0041] Shrubsole, K. , L. Worrall , E. Power , and D. A. O'Connor . 2019. “Barriers and Facilitators to Meeting Aphasia Guideline Recommendations: What Factors Influence Speech Pathologistsʼ Practice?” Disability and Rehabilitation 41, no. 13: 1596–1607. 10.1080/09638288.2018.1432706. 29376450

[jlcd70292-bib-0042] Speech Pathology Australia . 2022. Telerehabilitation in Speech Pathology. https://speechpathologyaustralia.org.au/SPAweb/Members/Position_Statements/Position_Statements.aspx?hkey=b1a46941‐246c‐4609‐bacc‐1c1b5c52d19d#tp.

[jlcd70292-bib-0043] Stephenson, A. , S. Howes , P. J. Murphy , et al. 2022. “Factors Influencing the Delivery of Telerehabilitation for Stroke: A Systematic Review.” PLoS ONE 17, no. 5: e0265828. 10.1371/journal.pone.0265828. 35544471 PMC9094559

[jlcd70292-bib-0044] Thomas, D. C. , R. Sutherland , N. Munro , et al. 2025. “Telehealth and in‐Person Placements: Same, Same, but Different. A Mixed Methods Investigation of Speech and Language Therapy Studentsʼ and Practice Educatorsʼ Experiences and Perceptions.” International Journal of Language & Communication Disorders 60, no. 2: e70009. 10.1111/1460-6984.70009. 39977726 PMC11842016

[jlcd70292-bib-0045] Tovin, M. M. , and M. E. Wormley . 2023. “Systematic Development of Standards for Mixed Methods Reporting in Rehabilitation Health Sciences Research.” Physical Therapy 103, no. 11: pzad084. 10.1093/ptj/pzad084. 37672215

[jlcd70292-bib-0046] Vuong, G. , C. L. Burns , J. Dignam , D. A. Copland , H. Wedley , and A. J. Hill . 2024. “Configuration of a Telerehabilitation System to Deliver a Comprehensive Aphasia Therapy Program via Telerehabilitation (TeleCHAT): A Human‐Centred Design Approach.” Aphasiology 39, no. 1: 93–124. 10.1080/02687038.2024.2314328.

[jlcd70292-bib-0047] World Health Organization . 2001. International Classification of Functioning, Disability and Health, ICF. World Health Organization.

[jlcd70292-bib-0048] Worrall, L. E. , A. D. Rodriguez , J. K. Dignam , et al. 2024. “A Modified Intensive, Comprehensive Aphasia Program (mICAP) has Better Reported Outcomes Than Usual Care: A Randomized Controlled Trial.” Aphasiology 39, no. 10: 1341–1360. 10.1080/02687038.2024.2424493.

