# Supplementary material for: Training for the Delivery of a Comprehensive High‐Dose Aphasia Therapy Program via Telerehabilitation: Effectiveness and Satisfaction With the TeleCHAT Training Package
Source: Int J Lang Commun Disord. 2026 Jul 14;61(4):e70292. doi: 10.1111/1460-6984.70292 (PMC13366443; doi:10.1111/1460-6984.70292)
Supplement: Supplementary file 1 — Supporting Information: jlcd70292‐supp‐0001‐SuppMat.docx [file JLCD-61-0-s003.docx]

**Demographic survey**

The purpose of this survey is to collect demographic data about your experience a speech pathologist and if applicable, your experience with telerehabilitation.

For checkbox questions, please tick your response.

Please write your responses for all other applicable information.

Note: an asterisk (*) denotes a required response.

**QUESTIONS**

1. What is your gender?*

- Female
- Male

1. What year did you gain your speech pathology qualification?*
2. What is your highest level of qualification?*

- Bachelor
- Bachelor with Honours
- Masters
- PhD
- Post-doctorate

1. Have you had experience delivering aphasia therapy before?*

- Yes
- No
  1. If yes, how long you work in this area?

Years Months

- 1. If yes, what type of therapy did you deliver?

1. One-on-one or group?
2. Impairment/Functional/Computer-based?
3. List all therapy activities e.g., semantic feature analysis:
4. Prior to training, had you delivered any aphasia therapy **via telerehabilitation** before?*

- Yes
- No
  1. If yes, what type of therapy did you deliver?
     1. One-on-one or group?
     2. Impairment/Functional/Computer-based?
     3. List all therapy activities e.g., semantic feature analysis:

1. Prior to the training, had you used telerehabilitation for delivery of other services before?

- Yes
- No
  1. If yes, what therapy did you deliver?
     1. One-on-one or group?
     2. Population:
     3. Area of speech pathology:
  2. If yes, what was your experience using telerehabilitation?

1. Prior to the training, had you used any videoconferencing platforms (e.g., Skype, Zoom, Facetime) before?*

- Yes
- No
  1. If yes, what purposes did you use theses platforms for?

1. Prior to the TeleCHAT training, have you attended any other upskilling or professional development workshops related to telerehabilitation?*

- Yes
- No
  1. If yes, what training workshop(s) did you attend?
  2. If yes, what skills/learnings did you take from the workshop?
