# Supplementary material for: Training for the Delivery of a Comprehensive High‐Dose Aphasia Therapy Program via Telerehabilitation: Effectiveness and Satisfaction With the TeleCHAT Training Package
Source: Int J Lang Commun Disord. 2026 Jul 14;61(4):e70292. doi: 10.1111/1460-6984.70292 (PMC13366443; doi:10.1111/1460-6984.70292)
Supplement: Supplementary file 2 — Supporting Information: jlcd70292‐supp‐0002‐SuppMat.docx [file JLCD-61-0-s002.docx]

**Observation of Technological Skills Checklist adapted from Hill & Breslin (2016)**

| **Task** | **Not at all independent** | **Moderately independent** | **Totally independent** | **Referred to training material** | **Required direct support** | **N/A** |
| --- | --- | --- | --- | --- | --- | --- |
| **Set up and use computer** | | | | | | |
| Connect internet |  |  |  |  |  |  |
| Connect headset/earphones |  |  |  |  |  |  |
| Connect microphone |  |  |  |  |  |  |
| **Task rating** |  |  |  |  |  |  |
| **Set up and use iPad/Tablet** | | | | | | |
| Connect internet |  |  |  |  |  |  |
| Connect and enable headset/earphones for videoconferencing app |  |  |  |  |  |  |
| Connect and enable microphone |  |  |  |  |  |  |
| **Task rating** |  |  |  |  |  |  |
| **Therapy preparation** | | | | | | |
| Access storage drive to find templates |  |  |  |  |  |  |
| Modify templates as per instructions |  |  |  |  |  |  |
| Download images sent by clients and add to resources |  |  |  |  |  |  |
| Open multiple documents/windows on second monitor |  |  |  |  |  |  |
| **Task rating** |  |  |  |  |  |  |
| **Set up videoconferencing (VC) software** | | | | | | |
| Open and login to VC software |  |  |  |  |  |  |
| Test camera |  |  |  |  |  |  |
| Test microphone and speakers |  |  |  |  |  |  |
| Video-call participant |  |  |  |  |  |  |
| Admit participant |  |  |  |  |  |  |
| Send participant meeting link |  |  |  |  |  |  |
| **Task rating** |  |  |  |  |  |  |
| **Within therapy activities** | | | | | | |
| Share screen of applications |  |  |  |  |  |  |
| Share screen of document camera/iPad |  |  |  |  |  |  |
| Share portion of the desktop |  |  |  |  |  |  |
| Share screen of internet browser |  |  |  |  |  |  |
| Share computer sound |  |  |  |  |  |  |
| Connect iPad as another user |  |  |  |  |  |  |
| Request for remote control of participant’s screen |  |  |  |  |  |  |
| Request for remote control of clinician’s screen |  |  |  |  |  |  |
| Remove participant’s ability to remote control |  |  |  |  |  |  |
| Find the Annotation tools |  |  |  |  |  |  |
| Draw, highlight, type, drag and indicate on **Whiteboard** |  |  |  |  |  |  |
| Draw, highlight, type, drag and indicate on **shared screen** |  |  |  |  |  |  |
| Type in the **Chat** box |  |  |  |  |  |  |
| Mute and unmute participant |  |  |  |  |  |  |
| Toggle view of participants (e.g., grid. Vs speaker vs pinned speaker) |  |  |  |  |  |  |
| Enable and type closed captions |  |  |  |  |  |  |
| Record video/audio |  |  |  |  |  |  |
| **Task rating** |  |  |  |  |  |  |
| **Finish therapy session** | | | | | | |
| End meeting for all |  |  |  |  |  |  |
| Send participant resources |  |  |  |  |  |  |
| **Task rating** |  |  |  |  |  |  |
