# Supplementary material for: Training for the Delivery of a Comprehensive High‐Dose Aphasia Therapy Program via Telerehabilitation: Effectiveness and Satisfaction With the TeleCHAT Training Package
Source: Int J Lang Commun Disord. 2026 Jul 14;61(4):e70292. doi: 10.1111/1460-6984.70292 (PMC13366443; doi:10.1111/1460-6984.70292)
Supplement: Supplementary file 3 — Supporting Information: jlcd70292‐supp‐0003‐SuppMat.docx [file JLCD-61-0-s001.docx]

**Satisfaction survey**

This survey aims to evaluate the TeleCHAT training.

Please take the time to think about each question and respond about your experience with the TeleCHAT training session.

To what extent do you agree with the following statements? Please circle your answers.

| **Statements** | **Strongly Disagree** | **Disagree** | **Neither Agree nor Disagree** | **Agree** | **Strongly Agree** | **N/A** |
| --- | --- | --- | --- | --- | --- | --- |
| 1. The TeleCHAT program has been explained to me. | 1 | 2 | 3 | 4 | 5 | n/a |
| 1. I understand all the components of TeleCHAT. | 1 | 2 | 3 | 4 | 5 | n/a |
| 1. I understand what is required of me to deliver TeleCHAT. | 1 | 2 | 3 | 4 | 5 | n/a |
| 1. The simulation session helped me understand how to use the computer and videoconferencing software (telerehabilitation system). | 1 | 2 | 3 | 4 | 5 | n/a |
| 1. I am confident using the telerehabilitation system. | 1 | 2 | 3 | 4 | 5 | n/a |
| 1. The mock technical difficulty procedure helped me understand how to troubleshoot any technical difficulties experienced. | 1 | 2 | 3 | 4 | 5 | n/a |
| 1. I am confident that I can troubleshoot any technical difficulties experienced from my end. | 1 | 2 | 3 | 4 | 5 | n/a |
| 1. I am confident that I can help the PWA troubleshoot any technical difficulties they may have on their end. | 1 | 2 | 3 | 4 | 5 | n/a |
| 1. I know that I can refer to the TeleCHAT manual to help me resolve technical difficulties. | 1 | 2 | 3 | 4 | 5 | n/a |
| 1. I know the chain of contacts to refer any technological issues to. | 1 | 2 | 3 | 4 | 5 | n/a |
| 1. I am confident that I can deliver all the components of TeleCHAT over telerehabilitation. | 1 | 2 | 3 | 4 | 5 | n/a |
| 1. There was a suitable mix of presentation and practical sessions. | 1 | 2 | 3 | 4 | 5 | n/a |
| 1. The training session has prepared me well for delivering TeleCHAT. | 1 | 2 | 3 | 4 | 5 | n/a |

Please answer the following questions in the text box below:

1. Is there any other information not covered in the training that you would like to know?
2. Do you have any suggestions to improve the training?
